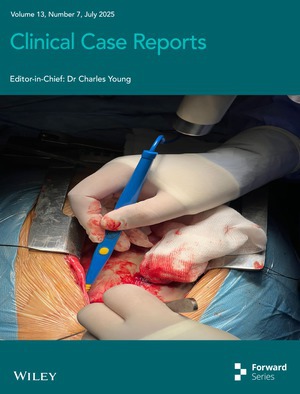# Cover Image

**DOI:** 10.1002/ccr3.70650

**Published:** 2025-07-16

**Authors:** Syed Mohsin Raza Bukhari, Muhammad Shahzad Raza, Muhammad Ans, Umema Shanza, Jalil Abbas, Kamran Yousuf, Mohsin Raza

## Abstract

The cover image is based on the article *Giant Aortic Root Aneurysm in a Young Patient With Marfan Syndrome: A Clinical Image* by Syed Mohsin Raza Bukhari et al., https://doi.org/10.1002/ccr3.70597.